# Insights from older adults’ lived experience of physical activity and exercise during the COVID-19 lockdown in England

**DOI:** 10.3389/fspor.2024.1395471

**Published:** 2024-10-31

**Authors:** Zsofia Szekeres, Noelia Agustín-Sierra, Lisa Zaidell, Katya N. Mileva, Rita F. De Oliveira

**Affiliations:** ^1^ School of Sport and Health Sciences, Cardiff Metropolitan University, Cardiff, United Kingdom; ^2^ School of Applied Sciences, London South Bank University, London, United Kingdom; ^3^ Political Science and Sociology, Complutense University of Madrid, Madrid, Spain

**Keywords:** barriers, motivation, government restrictions, walking, socio-ecological model, qualitative, psychology, neighbourhood

## Abstract

**Introduction:**

This study investigated older adults' emotional and social experiences of physical activity and exercise during the first Covid-19 lockdown in England.

**Methods:**

Participants were 24 older adults (M = 74 years, SD = 5.0) either physically active or inactive before lockdown. Semi-structured interviews were conducted at the start of the pandemic in England, and when restrictions were lifted eight weeks later.

**Results:**

Template analysis revealed three main themes: a Sense of Purpose for Being Physically Active, Routes for Engagement, and Inactive by Force? with different sub-themes for active and inactive participants. The lockdown restrictions emphasised the need to keep physically active in both active and inactive participants, but they perceived barriers differently. Both active and inactive participants found a renewed sense of purpose in walking outdoors for exercise when restrictions eased, enhancing their physical and mental well-being.

**Discussion:**

To reduce barriers and emphasise that exercise is for all ages and all levels of mobility, multi-modal recommendations are presented for educating, promoting, supporting, and enabling older adults to engage in physical activity and exercise.

## 1 Introduction

Global physical inactivity is prevalent, notably among UK older adults, with 27% of 55–74-year-olds and 47% of 75–84-year-olds doing less than 30 min of physical activity weekly (1). During the global COVID-19 pandemic, the UK enforced a nationwide lockdown in spring 2020, leading to “forced inactivity” for older adults, particularly the clinically vulnerable, urban residents, and those attending community-based exercise classes. This may have worsened the ongoing “physical inactivity pandemic” (2, 3), as several studies reported reduced physical activity among older adults during the pandemic (4–7). Social contact restrictions also significantly impacted the physical and mental well-being of both younger and older adults (8–12). This study aims to better understand the factors influencing physical activity and exercise engagement among older adults in the context of the COVID-19 pandemic.

The barriers to physical activity and exercise engagement have most often been assessed in cross-sectional studies and a limited number of qualitative studies (13) that found many intrapersonal and some interpersonal and environmental factors (14–19). These studies highlight that individuals’ engagement depends on themselves as well as their social and physical surroundings (for an extensive review of correlates and determinants of exercise framed by an ecological model (20). Ecological models (21–23) help to contextualise individual behaviour by considering factors at the intrapersonal, interpersonal, organisational, environmental and policy levels [e.g., (24–26)]. Because they are defined as metaconcepts (rather than theories), research has often paired them with another behaviour change theory (27). For instance, the self-determination theory (28) and in particular, the basic psychological needs theory (29), have been used as a theoretical framework to reflect on how the intrapersonal experiences of older adults may (de)motivate them to exercise by affecting their sense of autonomy, competence and relatedness. Briefly, the theory posits that the fulfilment of the three fundamental psychological needs leads to optimal functioning and well-being, and facilitates sustained engagement in an activity (30–32). Conversely, if these needs are not met, motivation to participate will be diminished (33, 34).

The lockdown led to disruptions in people's lifestyle habits and physical activity patterns (9, 10, 35). Although the UK Government permitted outdoor exercise for up to an hour daily, several key exercise facilitators, including professional support (36), a supportive environment, and social connections (36–38), were no longer accessible and were difficult to replace. Older adults changed their physical activity patterns during the pandemic (4, 5, 9, 11, 12, 39). Understanding their experiences is crucial for developing strategies to encourage (re)engagement (6). There is limited knowledge regarding the underlying multi-level factors that impact motivation to exercise in inactive older adults, for instance, how specific psychological barriers and health beliefs influence exercise participation, or how the built environment and types of social support foster motivation to exercise (40, 41). The main objective of this study was to explore older adults’ emotional and social experiences of physical activity and exercise before and during the Covid-19 lockdown, as revealed by both active and inactive older adults.

## 2 Materials and methods

### 2.1 Philosophical approach

In line with our aims to better understand the multi-level factors that influence older adults’ engagement in exercise and physical activity, we situated this research within a critical realist position framed by ontological realism and epistemological constructivism. A realist ontological position accepts that there is a real world with causes and effects that exists independently of our awareness. It is possible to access portions (42, 43) of that reality but only through what Fletcher (44) called theory-laden lenses. Our epistemological constructivism meant that we treated the participants’ discourse as a reflection of their values, experiences, beliefs, and behaviours. We acknowledged that participants had dispositions and experiences before they became participants (45), which were influenced by their participation in the study and by the lived context of the Covid-19. These underpinnings guided us in (a) designing the interview questions based on previous evidence and the socio-ecological model yet conducting the interviews flexibly to consider individual experiences; (b) analysing the data through an iterative process (see detail below); and (c) presenting participants’ quotes and explanations in the results to show results as a third-person account, staying close to their discourse.

### 2.2 Participants

Twenty-four participants took part in the study who were over 65 years of age (M = 74 years, SD = 5.0; 17 female and 7 male) and resided in Greater London (Table 1). We aimed to recruit at least 20 participants, following the recommended sample size (46) and based on qualitative studies in well-being and physical activity that reached data saturation with a sample of 12–22 interviews (46–50). We recruited participants by advertising the study via notice boards in several public places (prior to the enforcement of lockdown restrictions), and by email or telephone contact to community centres, older adult day services, and elderly organisations, who then advertised the study to older people. Inclusion criteria for study participation were: living independently, being cognitively able to participate, being aged 65 years or older and self-reporting not doing sufficient physical activity under the recommended level (51) in the two weeks before recruitment (see also Study design). This means that some participants had been sufficiently active before lockdown (hereafter called *Active*), while others had not (hereafter called *Inactive*), according to the current guideline^1^ (51).

**Table 1:** Socio-demographic characteristics and self-reported physical activity of participants.

		Inactive participants (*n* = 10)		Active participants (*n* = 14)	
Mean Age		76.2 years	SD = 5.9	72.4 years	SD = 3.7
Gender	Female	6	60%	11	79%
	Male	4	40%	3	21%
Ethnicity	White British	10	100%	8	57%
	White other origins	0		4	29%
	Asian	0		1	7%
	Black African	0		1	7%
Marital status	Single	1	10%	2	14%
	Widow, divorced or separated	5	50%	6	43%
	Married	4	40%	6	43%
Level of education	GCSE	3	30%	3	21%
	Diploma	2	20%	5	36%
	Degree	5	50%	6	43%
Work status	Retired	6	60%	8	57%
	Volunteer	3	30%	5	36%
	Employed	1	10%	1	7%
Health conditions^a^	Total mean, SD	3.5	SD = 2.1	1.8	SD = 1.3
Clinically vulnerable^b^	Total	5	50%	5	37%
Activity index before lockdown (MET)^c^	Total mean	553.8	SD = 225.0	1,205.3	SD = 543.4

^a^ Health conditions—the number of physical health conditions reported by the participants.

^b^ During the COVID-19 pandemic.

^c^ Metabolic equivalent.

From the 24 participants recruited, 10 (42%) were categorised as Inactive, and 14 (58%) as Active at the time of the first data collection point (Table 1). Participants’ physical activity level was based on the activity index as measured on the International Physical Activity Questionnaire—Short Form (53). The participants provided informed consent. The study gained ethical approval (ETH1920-0142) and was conducted per the Declaration of Helsinki.

### 2.3 Study design

This qualitative study used a framework-driven interview guide conducted in three moments: (retrospectively before the lockdown, during lockdown, and after the lockdown eased). After the first data collection point, we briefly informed all participants about online exercise opportunities via email or text message, in order to minimise “lack of knowledge” as a barrier and to provide an opportunity for all participants to engage in exercise. Collecting data at three time points allowed the researchers to gain a more comprehensive understanding of the changes in participants’ lived experiences of physical activity and exercise as they endured and adapted to the circumstances imposed.

### 2.4 Data collection

**Figure 1:**
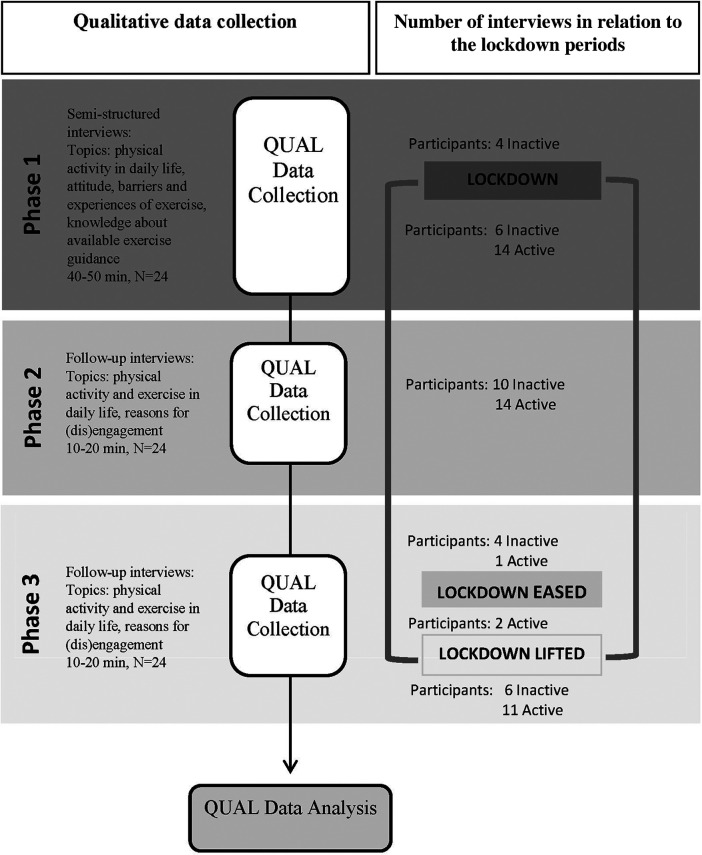
Participants flow and data collection in Phase 1, 2 and 3.

The study ran in three phases. Phase 1 was the baseline data collection and included interviews and the International Physical Activity Questionnaire—Short Form. Phase 2 was the first follow-up at 2 weeks and included interviews over the telephone. Phase 3 was the second follow-up at 8 weeks and included interviews and the International Physical Activity Questionnaire—Short Form over the telephone to reassess the physical activity and exercise engagement after 8 weeks (Figure 1). This timeline was appropriate for capturing potential changes in discourse and experiences as lockdown restrictions evolved. The first author collected all the data. Data saturation was achieved after analysing 22 interview data but the data of all 24 interviews was included in the analysis. During the study, the lockdown restricted personal contact between households, and no community exercise programs were active. Before the lockdown began in Phase 1, four participants were interviewed in person and filled out the International Physical Activity Questionnaire—Short Form on paper, while the remaining participants completed the interviews and the questionnaire via telephone.

#### 2.4.1 Interviews

##### 2.4.1.1 Phase 1

To measure the self-reported engagement in physical activity over the previous week and before the lockdown (including its intensity, duration and frequency), participants completed the International Physical Activity Questionnaire—Short Form prior to the interview. This questionnaire is a robust measure of physical activity within able-bodied populations. The semi-structured interview guide included questions about physical activity habits, barriers and facilitators of exercise participation under normal circumstances, as well as current physical activity patterns, facilitators and barriers under lockdown restrictions. In line with the socio-ecologic model of health (54), the questions aimed to cover different levels of influence, from intrapersonal (physical, cognitive, emotional), and interpersonal, to some aspects of the environment and organizational levels. The interview guide included both open and closed questions (see Supplementary Table S1). Three independent professionals working with older adults, reviewed the interview guide to ensure that the questions were relevant and clear.

##### 2.4.1.2 Phase 2

The short, semi-structured telephone interview included questions about participants’ emotional well-being and day-to-day life during the lockdown period and whether they had taken up any exercise.

##### 2.4.1.3 Phase 3

Participants repeated the International Physical Activity Questionnaire—Short Form over the telephone and participated in a semi-structured telephone interview, which included the same questions as in Phase 2 and asked about the well-being and exercise engagement as the lockdown was eased. Additionally, the researcher asked the participants if they had felt supported by any organizations, the local authorities, and the national Government to stay physically active over the previous two months.

### 2.5 Data analysis

We transcribed all interviews verbatim and analyzed the data using the computer-assisted qualitative analysis software NVivo Pro (QSR International Pty Ltd., 2020, released in March 2020). We chose template analysis, utilizing hierarchical coding in six steps, as listed in Supplementary Table S2^2^ (55). Template analysis, a pragmatic form of thematic analysis, is widely used in applied research due to its accessibility and actionability for practitioners (55). This method allows researchers to capture important theoretical concepts using *a priori* themes (55). Since *a priori* themes are not fixed, new themes can be identified and modified as needed (55). Therefore, template analysis informed the aims of the study and helped structure the findings to address practical concerns of practitioners. We used this approach to provide recommendations for practitioners and stakeholders who work with older adults.

The research team made several decisions to increase the rigour of the study. Firstly, the interviews began with simple descriptive questions to establish rapport with the participants and put them at ease before asking more exploratory questions about their motivation to exercise. Secondly, template analysis allowed for flexibility in coding enabling the refinement of the template as new themes emerged. This iterative process ensured that the template accurately reflected the data. Thirdly, selected quotations demonstrated that the findings and the conclusions were based on the data, helping readers reflect on the researchers’ interpretations (56). Finally, the final template was developed after consulting with two “critical friends” throughout the analysis (56). Two researchers developed the template by independently coding a subset of eight interviews. The primary researcher then coded the rest of the data but had the opportunity to reflect on her findings through several meetings with the other researchers. These discussions helped revise themes and subthemes, confirming that the interpretations were based on the participants’ experiences and perceived reality related to engagement in physical activity and motivation to exercise. During these meetings, the researchers also monitored whether coding and the development of new subthemes had reached data saturation. After analysing 22 baseline interviews, the researchers confirmed that no new subthemes or issues of interest were found. At that time, 24 interviews had already been completed, so all 24 interviews were analysed, and data collection was concluded with 24 participants.

## 3 Results

Our findings are presented in separate sections related to inactive and active participants. The organising themes were: a Sense of Purpose for Being Physically Active, Routes for Engagement, and Inactive by Force? (see Table 2).

**Table 2:** Main themes and sub-themes for the inactive and active participants.

Themes	Inactive participants	Active participants	Time
Sense of Purpose for Being Physically Active (barriers for inactive participants but the main facilitator for active participants)			Before the, lockdown
	Lacking interest or not seeing the benefits of exercise Limitations in physical competence Lacking social interaction	Health enjoyment and friends	
Routes for Engagement (facilitators for both groups)			
	Finding a purposeful activity Being aware of the need for exercise Convenience	Being aware of the need for exercise Strategies to complete unpleasant tasks Convenience	
Inactive by Force? (unexpected facilitator for inactive participants)			During and after the lockdown
	Staying physically active at home Starting points	Lacking social interaction Resilience in keeping up exercise	

### 3.1 Sense of Purpose for Being Physically Active

Lacking interest or not seeing the benefits of exercise was an important intrapersonal barrier for most of the inactive participants. Participants explained that they had difficulties finding a purpose for exercise, which was one of the main reasons they gave for lacking motivation. Inactive participants who had exercised before had not enjoyed the exercise or the setting, they never found it interesting and saw it as a “waste of time and money”. Some inactive participants did not believe in the benefits of exercise, felt no need for it, or felt that exercise was not for them due to their age.

I just want to continue being able to do my daily chores fine and survive. I have a few problems doing them, but I can still cook, dress myself. I am also not sure if those exercises would do me any good. (Inactive 72-year-old male, White British, at Phase 1)

The inactive participants tended to prioritise exercise much lower in their “to-do list”, and their discourse was often dominated by providing excuses or procrastinating the exercise.

But again, it is about motivation, which sort of mood I have in the morning. Because of the age, I am retired, so I have that choice, don't I, so I can do whatever I want to….Oh, the motivation [to go for a walk in the park], sometimes I just start doing something else, and I then forget. (Inactive 78-year-old female, White British, at Phase 1)

It is noteworthy that, for some inactive participants, exercise had been very important in the past. For example, they had active childhoods or had a favourite sport. Then, however, they gave it up due to injury, competing responsibilities in adulthood, and lacking the confidence to re-engage.

I think it is a matter of where my head is and where my heart is at the moment. I just don't have the enthusiasm for spinning that I used to have. I lost confidence somehow. (Inactive 79-year-old female, White British, at Phase 1)

Limitations in physical competence was the other intrapersonal barrier that was mentioned by some inactive participants and these were related to health problems, for example heart problems or cancer.

Probably now I could do some exercise. I just didn't really get back to it. But maybe the other thing that stops me is that I know I would tire more quickly now, and I get out of breath more quickly. (Inactive 79-year-old female, White British)

Limitations in mobility, increased pain or experience of falling stopped several participants from doing certain types of exercise they had enjoyed in the past. Some of them felt that alternative exercises would be too vigorous, not interest them, or not be enjoyable for them.

And when I tried Tai Chi, I felt a bit embarrassed because I couldn’t do all the exercises. I have a bad knee so I couldn’t stand on one leg. (Inactive 67-year-old male, White British, at Phase 1)

Due to cognitive and emotional challenges, psychological limitation was identified as another significant barrier to exercise by inactive participants. Participants felt that it was a “mental thing”, that “something has changed” and they did not feel like doing exercise anymore, or they “got a bit down” and became “anxious about it”, particularly after experiencing physical health problems. Only a few inactive participants mentioned feeling self-conscious about body image and difficulty concentrating, while low motivation and lack of self-discipline came up more frequently. Lacking social interaction to exercise was an interpersonal barrier that made some inactive participants lose their interest and struggle to find purpose in the exercise class. For example, one participant stopped dancing when their friend could not join her anymore: “so it is harder to get motivated”. For those without a strong drive to exercise, recommendation from a health professional, social support from friends, family, or an exercise group could perhaps be the most motivating factor.

If someone would come with me, I think. I was actually talking with my partner about it and thinking that we could go to the leisure centre together. But he works until 5 every day and after he is tired of course. And on the weekends, we have other things to do. (Inactive 67-year-old male, White British, at Phase 1)

Health enjoyment and friends were the main purposes that helped active participants stay active. The initial drive to exercise for most active participants was maintaining health and physical fitness. They appreciated that these exercises allowed them to “be able to walk, doing everything by (themselves)” and having no “need for any help from others; so doing the exercises helps a lot”. Socializing was also a key purpose of exercise as well as finding an activity that suited their physical needs. Many active female participants shared their experiences of joining a gym or exercise group with a friend.

My friend told me about it (Aqua aerobics) and then I started to go with her. I cannot swim, but I gave it a try and found it very good. First, I was a bit unsure, a bit afraid of it. But once I started, I just stuck to it, I found it really beneficial, and I go on my own now. (Active 71-year-old female, Black African, at Phase 1)

Some of the active participants reported that having an incentive, such as losing weight or doing a challenge, motivated them to start an activity in the past but in order to adhere they needed to find the activity enjoyable or perceive its benefits, for example feeling “lifted and more energetic” after a class or managing arthritis. Those who attended group-based exercises believed that “joining a class is always helpful and motivating” because “it is a nice social activity as well.” On the other hand, individual exercise may be less likely to build a sense of community:

At the swimming club, I wouldn’t mix with the people for the rest of the week, I would just meet them there, they come from all over [the city]. Sometimes we have a coffee but most of the time people just come and get back home. (Active 68-year-old female, White Irish, at Phase 1)

To summarise, all participants needed a sense of purpose to engage in their leisure activities. Positive experiences and interpersonal factors during exercise helped active participants adhere, while lacking interest, social support, or a sense of competence were barriers for inactive participants to find a purpose in exercise. Their daily life physical activity was facilitated by interpersonal and environmental factors.

### 3.2 Routes for Engagement

Finding a purposeful activity was an important route that helped inactive participants to engage in some form of physical activity. The most popular physical activity for them was walking, not as an exercise but as part of daily life: “not doing necessarily a lot of physical exercises like touching my toes or anything like that, but I think walking is one of the best exercises one can do.” Other activities such as volunteering, working, being a grandparent or a carer were identified as an important part of participants’ daily lives, which provided an occupation and a structure to their days and often represented a part of their identity.

A few inactive participants who had done exercise before explained how important it is to find an exercise activity that matches their health needs and physical abilities, so it can give them a sense of accomplishment. However, the class would also have to be social or meet their interests as it can be “very well recommended but if it is not your sort of thing, you won't stick with it”.

Dancing was good fun, we put some shows on. And there was an end result. I think that is what I like, so you can see you achieved something. And that was nice and sociable as well. (Inactive 78-year-old female, White British, at Phase 1)

Being aware of the need for exercise was identified as another potential intrapersonal route for inactive participants. Maintaining physical function was often mentioned, for instance, “as I get older [exercise] would help get around better and have less problem with falling over”. One might take up exercise for some time; for example, a participant with diabetes thought: “I have to do something, it was a wake-up call”, but with time they stopped exercising. On the other hand, even specific advice on physical activity from the general practitioner was not sufficiently motivating for other inactive participants, as one of them expressed.

My GP is always telling me that I should exercise, but I wouldn’t do it even if I am told, that doesn’t work for me. I need to be interested in it. (Inactive 72-year-old male, White British, at Phase 1)

The importance of keeping active was rated highly for most inactive participants; however, they understood it as a need for maintaining their mobility as they age. One of them highlighted, “when I damaged my Achilles tendon that was shocking, I was unable to do things, so it is important to keep active”. On the other hand, most of the inactive participants did not acknowledge a strong link between doing regular cardiovascular, strengthening or balance exercises and “keeping active and healthy in old age”.

Convenience was the third route to exercise for inactive participants. This was an environmental factor mainly related to the location of exercise places. Participants expressed a preference for joining a gym or exercise group in their local area; “somewhere in walking distance” or at around 20 min bus journey in their close neighbourhood. “If there was a gym at the bottom of my stairs, I would be in the gym all the time.” On the other hand, most participants knew a local leisure centre that they could access but the proximity of an exercise opportunity was insufficient on its own.

But I shouldn’t really have any excuses because we have a big leisure centre right next door virtually. But I don’t want to do any of the group activities, I don’t think. (Inactive 87-year-old female, White British, at Phase 1)

Regarding affordability, inactive participants often stated that they “wouldn't feel comfortable paying” for exercise. Instead, they felt it should be part of an exercise referral scheme or free given their age and pensioner status.

Being aware of the need for exercise was the most often cited intrapersonal factor for active participants. They particularly highlighted the importance of active leisure as part of their weekly activities. Before the lockdown, they regularly attended group-based exercise classes or went to the gym. For many, exercise was part of their identity: “For me, it is very important, I have been active from a very young age, my family was very sport-oriented and then I carried that on from a child until now really.” Active participants shared strategies they used during exercise. For example, some set a target or had a routine, and others used distractors: “I sing while I am doing it”. Other participants only did the exercises they felt they could do or had an “emergency plan” in place like a chair nearby if they felt too fatigued during exercise.

Strategies to complete unpleasant tasks were important for active participants to maintain exercise. They rarely mentioned barriers to regular exercise (apart from barriers experienced after the lockdown described later) and found alternative ways to keep exercising. For example, participants who had had injuries or a history of falls reported that it may have reduced their confidence or made them stop certain types of exercise. Still, they took up exercise alternatives better suited to their needs. Changing circumstances were identified by some active participants as potential barriers, but they had the confidence to overcome those. For example, some of them changed their exercise class because they suddenly had a new responsibility in their lives. Others mentioned other priorities as the reason for occasionally missing a weekly exercise class which they replaced by doing more walking or another exercise class during the week.

Convenience was sometimes important for active participants, but it was not identified as a barrier. Some attended their favourite exercise class far from home, and they even took the train or car to get there. Others paid yearly membership in the local gym for the convenience of proximity. Other active participants went to their local leisure centre or park; they preferred not to travel further and preferred to pay session by session anything between £2 and £10.

To sum up, both inactive and active participants valued physical activity and rated its importance highly, but they had different interpretations of what it meant to be physically active. For inactive participants, being physically active meant keeping their mobility in old age. They expressed that interpersonal or environmental factors could motivate them to exercise. For active participants, being physically active meant doing regular exercise and keeping their sense of fitness and strength, so their motivation was mostly related to intrapersonal factors.

### 3.3 Inactive by Force?

Staying physically active at home was an unexpected motivator for inactive participants, who became more conscious of the need to be (physically or mentally) active in their daily lives. While inactive participants had been busy with home-based activities such as cooking and cleaning, the lockdown restrictions awoken a need to be more purposeful in their physical and mental pursuits. They started structuring their days to ensure they “achieved something,” so they did not “let things just go all day, without it it's easy not to bother.” They listed activities related to their environment, such as “gardening, tidying up”, a variety of housework chores or cooking and even larger home-based DIY jobs. Some included mental challenges such as using puzzles, crosswords or reading to keep their “brain active”. As the “novelty” of the lockdown wore off, boredom and lack of motivation became more prominent, and participants felt frustrated and “fed-up”. These negative feelings reduced their motivation to be (physically) active: “it is like the less you do, the less you want to do”. As a result, when the lockdown eased, participants were “excited about meeting friends and family” but were also enthused about going out for walks in their neighbourhood as a form of exercise (see next sub-theme).

Starting points for inactive participants were impacted by the environmental-level restrictions. They realised the importance of exercise to “stay active” and not let their “fitness level deteriorate, particularly during this lockdown”, and they felt that doing some form of exercise positively impacted their mood. Some started to do their physiotherapy exercises at home, started regular walks, or climbed up and down the stairs in their flat as a form of exercise. Having a target, large or small, supported some inactive participants to do physical activity during the lockdown, for example, “going for the newspaper” or completing a “[Couch to] 5 K challenge with the NHS”.

Inactive participants were not always aware of the guidelines for physical activity, and they appreciated the information given to them by the research team about the recommended exercise. One participant stated, “if it means that I [have] got to do 150 min of exercise per week I would do that yes. If that is proven that helps, then I would do that.” The follow-up interviews revealed that the few inactive participants who became more active during the lockdown did that mainly because they became more aware of their own physical activity levels and felt exercise could help with their physical function or physical health problems. Some took the information we gave them to re-start exercising.

I started doing it after I spoke with you, I’m doing the strengthening exercises for my knee on YouTube, there is a guy who does about 15–20 min exercise. And it is good because I can feel that it helps, my knee is not hurting that much when I walk on the stairs for example. (Inactive 69-year-old male, White British, at Phase 3)

Lacking social interaction was identified as a dominant interpersonal barrier to exercise during the lockdown for the active participants. Participants found that it was hard to get motivated and follow videos or use other resources to exercise independently, and they rarely found it enjoyable.

The difficulty is that you cannot get motivated. I feel that when you are consciously going for something it is very difficult to maintain. You can start doing it and then it phases out, isn’t it? (Active 69-year-old female, White Croatian, at Phase 3)

At the follow-up, these participants were happy that their exercise provider re-started their (outdoor) exercise sessions and they could book their place. In addition, for many active participants having company or support from a friend or family member was a strong motivator for them to go for a walk, which was an exercise that allowed social distancing. Some active participants did use exercise videos, virtual live sessions, or repeated exercises from memory. The videos were chosen because the instructor was the same as in their usual activity, or the exercise challenged them, or they felt its benefits, so they tried to do that regularly.

And the other thing I do daily is that I do the Tai chi exercise on YouTube that you also sent me. That is 36 min and I actually don’t need to do it sitting, I do that standing. My balance is good enough to do that standing. And I think actually it helped my balance by doing that. (Active 75-year-old female, White British, at Phase 2)

Resilience in keeping up exercise was strongly impacted by interpersonal and environmental factors, but intrapersonal facilitators made most of the active participants stay active during the lockdown. They kept up exercise because they wanted to maintain their good level of physical function, sense of fitness and strength. At the start of the lockdown, many of them perceived a reduction in their fitness because they were not training as intensively as before or were sitting more throughout the day. Some stated that they did not expect their fitness to improve during the lockdown “because of the difficult circumstances but definitely not to deteriorate”. They realised the importance of exercise even more as they were allowed to leave the house for exercise for one hour a day: “it is the time in your day when you feel human, you go out so it is very important to do exercise”. Those who maintained their exercise level experienced emotional benefits such as better mood, sense of energy and achievement. Structuring the day and managing weight by continuing to exercise was also appreciated.

It is keeping me a lot more bouncy. My body feels quite relaxed and smooth when I get up from my chair and do that sort of thing. I am sure that it does me good. Because you know it is very easy just to sit indoors doing nothing, but these sorts of things make you say: “ahh ok I do some of the exercise routines. So it gives me the motivation as well to do things and it keeps you fit. (Active 68-year-old female, White British, at Phase 2)

To overcome apprehension related to the risk of infection, most participants used specific strategies. For example, “getting up very early in the morning before anyone is around” to go for a walk, or telling themselves, “you used to go out a lot and you can't now, so you have to do something to get your body moving”. Establishing an exercise routine during the lockdown was very important to some active participants because they realised that “like washing teeth”, the routine of their weekly exercise sessions contributed to their health and structured their days. Therefore, establishing a new exercise or walking routine motivated them to keep exercising, especially if this involved another person.

My neighbour who is at similar age and she is very active, she decided that we should go on walks together keeping distance. I am 72 and my neighbour is 84, but very fit person. She is a very active person, Mrs Motivator, so she walks first and I am behind her. (Active 72-year-old female, White Polish, at Phase 2)

Environmental factors influenced the maintenance of exercise: technical difficulties in using their electronic devices, not finding enough space in their home, or finding the level of the instruction unsuited to their abilities. Also, those living in city flats perceived the lack of space as substantial barriers that negatively impacted their physical activity level and mood.

This lockdown is a problem, I used to go out every day and walk around to different places, but now you have to stay at home, and you cannot just walk around the kitchen all day, can you? So it has been distressing really for people who need to exercise. (Active 77-year-old female, Asian, at Phase 2)

With the easing of the lockdown, many participants raised their concerns about people not respecting the social-distance regulations and the crowdedness of the open spaces where they were walking and the streets where they went shopping. At the same time, seeing more and more people exercising in the wider community of the local area encouraged a few participants to follow suit.

When I go around the park, because everyone else is exercising, I also don’t mind. So I do sort of swings with my arms and things like this. As everyone else is exercising I don’t think that they would laugh at me because they do that themselves. (Active 68-year-old female, White British, at Phase 3)

Both inactive and active participants acknowledged the need for and tried to incorporate physical activity into their days during the lockdown but not all were successful. The main facilitators for being physically active were a routine, going outdoors for a walk alone or with some company, finding an exercise matching their needs and abilities, and living near green spaces. Outdoor physical activity was described as an important part of the daily activities which brought positive benefits to participants’ well-being.

## 4 Discussion

This study explored the factors that limit or support the engagement in regular exercise among older adults who resided in Greater London. A significant result was that finding a sense of purpose for exercise was the main facilitator which distinguished active from inactive older adults. Other facilitators of adherence to physical activity (or exercise) were establishing a routine, having social support and perceiving an immediate benefit from exercising, especially when there was no access to community-based exercise classes during the lockdown.

The sense of purpose for exercise was identified as a key element of exercise motivation in this study. It was found to be influenced by both the belief in the importance of exercise and the value participants assigned to exercise. The sense of purpose for exercise seemed to be missing from the perceptions of inactive older adults due to cognitive and affective processes. In terms of cognitive processes, being aware of the importance of maintaining mobility in later life did not lead to adherence to exercise. Participants saw their age as a barrier and thought the gym or intensive exercise was not for them. Importantly, they were unaware of how certain types of exercise could improve their strength and balance and help maintain an optimal level of physical function. In terms of affective processes, the sense of purpose when it comes to enjoyment had been lost through negative experiences in the past. For example, feeling pain or having physical limitations caused withdrawal from a favourite exercise or caused a lack of success in trying a new exercise. This reduced sense of competence and fitness led to them losing confidence and developing negative feelings about exercising. Therefore, the barrier is not only the physical limitation itself, as was reported in several previous studies (13, 40, 57), but what the physical limitation means to the individual. According to the Affective-Reflective Theory, experiences, feelings and thoughts connected with exercise can cause an automatic negative affective valuation which reduces the motivational drive towards exercise (58). The automatic valuation of a previous negative experience also serves as the basis for controlled, reflective evaluation in the present, and it can cause avoidance and negative anticipation about the consequences of exercise (58). The negative reflective evaluation could be linked with amotivation, and with not having one's basic psychological needs met. Ryan and Deci (29) describe the importance of the psychological needs for competence, relatedness and autonomy to build motivation (29).

Our results suggest that older adults who did not find a sense of purpose in exercise had previously had negative experiences of not having their basic psychological needs met in the context of exercise. The application of these theories to our findings helps us understand the reason why most of the inactive participants who have physical limitations do not exercise regularly despite valuing long-lasting mobility and independence. Therefore, older adults should be supported with a variety of strategies to regain confidence and a sense of purpose for exercise.

What factors contribute to gaining a sense of purpose in exercising? What can we do to support inactive older adults to have their basic psychological needs met in exercise contexts? Some facilitators of exercise can be derived from the active participants’ accounts, and some by combining them with inactive older adults’ barriers. Exercise facilitators are a combination of perceived immediate benefits to mental and physical health, social life and routine. The effect of the lockdown highlighted the importance of a daily routine for older adults. For most inactive participants, volunteering, caring or social leisure activities were things that structured their week before lockdown, while for active participants, exercise and physical activity provided routine. During the lockdown, active participants missed that routine and therefore struggled to adhere to home-based exercise. However, several participants reported that having a routine of walking or doing short exercise bouts positively impacted their mood and reduced their anxiety. This is in line with previous research which also found that routine is vital and can contribute to a sense of autonomy, improved well-being, and self-esteem after retirement (59). These findings indicated that a routine which includes exercise may provide an added sense of purpose and this should be promoted to inactive older adults.

Another factor contributing to gaining a sense of purpose is perceived immediate improvement in mental and physical health. During the COVID-19 lockdown, the increased external barriers to exercise and the restricted opportunities for outdoor activities had negative effects on participants’ mental health and made them more conscious about the importance of being physically active outdoors. Therefore, going out for a walk with the purpose to exercise increased the total weekly walking time for most participants. This included inactive participants who recognised the negative impact of being sedentary on their ease of mobility and mood. For all participants, walking provided a rare opportunity to leave their homes or to meet with their relatives and friends as the lockdown was eased. Participants reported the meaningful effect that walking outdoors had on their mental health which meant that they found a strong purpose for walking outdoors. Walking outdoors was also the preferred way of exercise compared to following online videos for active participants. A recent study on a home-based fall prevention exercise programme delivered online also found that many participants prioritised outdoor exercise more than tablet-supported exercise (60). Walking outdoors can serve the need for competence as it helps interact with the environment, and it can also challenge individuals to gain a mastery experience (29, 60). Taken together, these findings suggest that experiencing the immediate benefits of exercise might be an excellent way to engage inactive older adults, and walking as exercise may be the best starting point. Importantly, some inactive participants thought exercise was not for them (given their age and mobility problems) so informing them that exercise is beneficial for all ages and abilities is crucial.

The final factor that can contribute to gaining a sense of purpose is social connectedness. The lockdown meant that some of the active participants lost their sense of purpose in exercising because exercising at home, on their own, did not allow them to connect with others (59, 61). Missing the sense of relatedness during exercise resulted in a lack of enjoyment which seemed to cause similar psychological barriers that were experienced by inactive participants. Other studies also found that many older adults gave up fall-prevention exercises (62) and did not follow exercise videos at home in lockdown (63), because they lost the social ties to the exercise group. Similarly, the sense of relatedness was found to be an important factor of motivation in older adults (14, 64). Therefore, we suggest that it is important to allow participants to re-connect before or after the group exercise sessions to re-build those social ties within the group. Support should be available also for those who need to isolate for longer and for those who need to build their confidence up to return to a group. For instance, online exercise classes could stay on offer and smaller exercise classes with 4–6 people could be introduced before a larger class. For inactive older adults, strategies to engage them in exercise should consider the use of an exercise buddy. For example, strategies can capitalise on some communities having been brought closer together as a result of lockdown, or capitalise on the fact that some inactive older adults started walking with the company of a friend or family member.

Community-based exercise programmes, before the lockdown, provided active participants with a setting where not only their physical but also their psychological needs were met. In particular, active participants had felt competent by doing exercise tasks that were suited to their abilities and by feeling its benefits; they had felt relatedness by building meaningful relationships and feeling connected to others, and they had felt autonomy because they consciously took action to maintain or improve their physical function. In contrast, inactive participants shared their stories about previous failed attempts to take up exercise due to not having a sense of competence or not having company for exercise. Recent reviews suggest that interaction with peers is an important motivational factor for older adults, which facilitates enjoyment, drives exercise uptake and adherence (59, 61). Inactive participants were not able to realise the fulfilment of any of the three psychological needs in the context of exercise. Instead, these needs were met through other activities. For example, doing crosswords met their need for competence, visiting friends met their need for relatedness, and doing their shopping met their need for autonomy. Therefore, we suggest that for inactive older adults, interventions should focus on breaking down barriers to build a positive perception of exercise for example, through government-led media campaigns. To address the need for competence, we suggest that campaign messaging clarifies that exercise is for all ages and levels of mobility, and that exercise provision is adapted to the needs of older adults. To address the need for relatedness, we suggest that exercise is combined or fitted around other social activities that they already engage with and are in line with their interests. To address the need for autonomy, it is important that exercise provision offers choice and that it includes positive messaging around older people taking charge of improving their mobility.

The context of this study, which took place during a national lockdown, provided unique insights into the pathways for motivation to exercise. Both active and inactive older adults resorted to walking as the main source of exercise and reported immediate health benefits and a sense of achievement from doing so. Research has shown that walking outdoors is a significant source of physical activity and has several benefits for physical health, and emotional well-being (65, 66). Even when performed for a relatively short time or at a slow pace, walking has several health benefits (67, 68) requires no specific skills or equipment and is convenient and accessible to many people, therefore walking has been identified as the most efficient way of improving physical activity levels in older adults (69–71). Moreover, five of the inactive participants reported 150 min of low-intensity physical activity a week (pre-lockdown) by walking to shops or social activities, gardening and housework. These activities could be modified to become moderate-level intensity which would reclassify them as active. This emphasises the potential for walking and potentially other activities to transition older adults from inactive to active. Both active and inactive participants also often reported walking with a company as a source of commitment and enjoyment. There is scarce evidence on the effectiveness of commitment-making as a tool to increase adherence to physical activity and the available research reported no significant improvement (72). However, the studies included behavioural contracts rather than committing to a friend. While committing to someone is an extrinsic motive and a form of introjected regulation (73), it might be a stepping stone to improve motivation to exercise in inactive older adults. Based on our findings, we suggest that commitment-making to a friend might be a potential facilitator for exercise uptake which should be further tested in research trials. Furthermore, based on the strong evidence on social ties as a facilitator in older adults, the social opportunity during exercise should be emphasised as part of physical activity advice for inactive older adults. Together, these results indicate that walking (with a friend) may be the single best strategy to get inactive older adults to engage with exercise. This together with education about the benefits of exercise for all ages and levels of mobility could be the core of government-level communication that would be crucial for post-pandemic recovery. Despite the turmoil caused by the pandemic, these unusual circumstances provided precious insight into inactive older adults.

This study has several important limitations. First, the study sample was limited to older adults who self-reported their activity levels and did not measure their physical activity level objectively. Participants in this study may have been more aware of the benefits of physical activity and more motivated towards behavioural change than those who are sedentary and did not participate in the study. This study has a limited sample of very sedentary participants; therefore, we do not know whether the conclusions and recommendations of this study could be generalized to the wider population of inactive older adults. Future research might consider using gatekeepers, such as healthcare professionals, to identify very sedentary individuals based on the NICE guidelines following brief physical activity conversation and advice to patients and signpost them to the research. Other limitations are the limited demographic variability and uneven gender distribution in our sample. The limited demographic variability means we cannot address important factors such as ethnicity, disability or socioeconomic status. Although we intended to recruit varied demographics, the constraints of the lockdown posed additional barriers to purposive sampling and specific recruitment strategies to reach different demographics. A final limitation is that the majority of semi-structured interviews were completed over the phone, making it more difficult to establish rapport with the participants than in person and it may have impacted the depth of the information being shared.

To address this, we employed several measures to increase validity and reliability. Firstly, we used a comprehensive and standardized semi-structured interview guide, including predefined questions and prompts, to ensure consistency across all interviews as suggested in the literature (74). Secondly, the first author, who collected all the data, was sufficiently trained and experienced in conducting telephone interviews and well-being consultations as part of her professional role, allowing her to handle various respondent reactions and situations consistently (75). Thirdly, the research team held several discussions about the findings and analysis to challenge assumptions and interpretations during data analysis (76).

### 4.1 Conclusion and recommendations

This research provides insight into the nature of perceived barriers and facilitators for engagement in physical activity among both physically active and inactive older adults. The longitudinal qualitative approach, with follow-up telephone interviews at 2 and 8 weeks and reassessment of self-reported physical activity at 8 weeks, was useful in providing depth to the data interpretation. Our main results showed that all participants relied heavily on having a sense of purpose in their daily leisure activities, suggesting that finding a sense of purpose for exercising might help “unlock” amotivation in inactive older adults. Notwithstanding, we found that the main routes for engaging in exercise were similar for active and inactive older adults and included perceived benefits to mental and physical health, socialising and routine. Importantly, during the COVID-19 lockdown, both active and inactive participants found a renewed sense of purpose in walking outdoors for exercise. Our main recommendations are for educating, promoting, supporting, and enabling older adults to engage in physical activity and exercise (see in Table 3 and Supplementary Table S3 for concrete examples).

**Table 3:** Selected list of recommendations for the engagement of older adults in physical activity.

Area of recommendation	Recommendations
Educating	
Purpose linked with the benefits of exercise	Older adults can be educated about the benefits of exercise for all ages and levels of mobility, especially concerning mobility and emotional well-being.
Purpose linked with social interaction	Older adults can be advised that finding a friend or family member with whom they can exercise or go on regular walks can help them to take up exercise by providing accountability.
Promoting	
Being aware of the need for exercise	Government advertisements should include positive messages about physical activity in older adults and guidance.
Staying physically active at home	The Government should be aware that older adults are not sufficiently physically active at home (even if they try). There should be policies to incentivize older adults to go outside for physical activity.
Starting points of exercise	Promotion of the benefits of outdoor physical activity can encourage older adults to improve their physical activity. It is important to ensure the time and availability of public places where older adults can do outdoor activities safely.
Supporting	
Purpose linked with interest (or priorities)	Professionals working with older adults can assess their interests and priorities in daily life, and signpost them to local activities which meet their interests.
Purpose linked with social interaction	Older adults who like socialising can be signposted to team sports or group-based exercises which include an element of social gathering.
Purpose linked with social interaction	“Exercise befriending” can be provided over the phone where active older adults encourage inactive peers to do physical activity while under lockdown restrictions.
Starting points for exercise (in lockdown)	Organisations should endeavour to facilitate the contact between older adults who were previously engaged in group exercise as a way to re-engage the whole group back into group exercise sessions.
Purpose linked with physical competence	Exercise professionals working with older adults should continue tailoring exercise tasks to individual needs and physical abilities to improve confidence and provide a positive experience during exercise.
Purpose linked with physical competence	Healthcare professionals should give clear straightforward advice about the intensity and type of exercise that is suitable for individual older adults with long-term health conditions or health concerns.
Enabling	
Purpose linked with physical competence/confidence	Improving the labelling and the description of exercise programmes could help inactive older adults find a type of exercise that is most likely to suit their physical abilities and give them confidence from the start.
Convenient location	Open, safe, and well-maintained infrastructure can enable older adults to walk more and do physical activity and exercise outdoors. Therefore, safe walking paths in parks with benches at regular intervals and hygiene facilities should be made available.
Purpose linked with the benefits of exercise/interest	Interventions should target enjoyment and social aspects as well as improving physical function to increase uptake and adherence because older adults tend to appreciate these aspects when they attend exercise programmes.

## Data Availability

The anonymised transcripts supporting the results of this article will be made available by the authors upon request for research purposes only.

## Footnotes

^1^The World Health Organization recommends that for substantial health benefits older adults should participate at least 150 min of moderate-intensity aerobic physical activity; or 75 of vigorous-intensity aerobic physical activity; or an equivalent combination of moderate- and vigorous-intensity activity throughout the week. These activities should also include strengthening activities twice a week and varied multicomponent physical activity (for example strength and balance fall prevention exercises with weights and resistance or own body weight) that emphasizes balance on 3 or more days a week, to enhance functional capacity and to prevent falls (51, 52).

^2^Brooks J, King N. Repository qualitative psychology in the real world: the utility of template analysis. *Br Psychol Soc Annu Conf* (Unpublished). London: University of Huddersfield Repository (2012). p. 18–20. April.
