# Supplementary material for: Insights from older adults’ lived experience of physical activity and exercise during the COVID-19 lockdown in England
Source: Front Sports Act Living. 2024 Oct 31;6:1395471. doi: 10.3389/fspor.2024.1395471 (PMC11560469; doi:10.3389/fspor.2024.1395471)
Supplement: Supplementary file 1 [file Table1.docx]

# Table S1. Semi-structured interview guides at Phases 1, 2 and 3 including topics and questions related to the levels of socio-ecologic model (SEM, McLeroy, Bibeau, Steckler, & Glanz, 1988) and previous findings of the research team

| Interview guide at Phase 1  (before and during the lockdown) | | |
| --- | --- | --- |
| SEM Level | Areas to explore  *(Themes and sub-themes found in our previous research; Szekeres, 2022**)* | Questions |
| Intrapersonal | **Preferences in leisure activities past and present**   - *Being active at leisure time* - *Exercise history*   Roadblocks (past and present)   - *Changing circumstances* - *Lacking interest* - *Difficulty in getting out of comfort zone*   Starting point   - *Having a drive for exercise* - *Need for support* - ‘*Giving it a try*’ | Preferences   1. How do you spend your leisure time in general?   *(The activities could fit in one of these categories:*   - *Social/individual* - *Active/Inactive* - *Cognitively challenging/Repetitive)*   If you think about exercise what comes into your mind? Why?  What do you do to be physically active (if you do anything)?  How do you spend your leisure time in the current circumstances??   1. Have you tried any exercise in the past?   Which kind of exercise you did in the past you really enjoyed?  Why did you enjoy it?  What stopped you?  Roadblocks   1. What stopped you from doing exercise regularly before? 2. How the lockdown impacted on your physical activity and on the things you normally do? 3. How important is it to you to be active? (1-10) Why? 4. How confident are you that you can be more active? (1-10) Why?   Starting point   1. What would make you be more physically active?   *(For example:*   - *Thinking about physical/cognitive/emotional health* - *Socializing* - *Try something new* - *Being fit/lose weight* - *Schedule your day* - *Recommendation from a trusted person)*  1. What could help you to start? How did you start XY leisure activity?   Would you happy to start exercise now on your own? Or who could help you?   - *If needing support: friend/family/in a group/contact with someone initially*  1. How competent do you feel to give it a try? (1-10) Why? 2. What could help you to start? How did you start XY leisure activity?   Would you happy to start exercise on your own? Or who could help you?   - *If needing support: friend/family/in a group/contact with someone initially*  1. How competent do you feel to give it a try? (1-10) Why? |
| Intrapersonal | Mindsets - Thought processes and strategies   - *Overcoming negative thoughts* - *Being able to adapt* - *Looking forward to* it   Benefits of participation   - “*Gave me something back”* (relatedness and competence) - *Perceived health and well-being* | Mindsets - Thought processes and strategies   1. How do you cope with the changes caused by COVID19? 2. How do you keep active? 3. What helps you carry out a plan when you don’t feel like it? 4. What would you do if other appointment or responsibility need to be scheduled in?   (note: strong commitment to plan vs. strong commitment to pleasure)  Benefits of participation   - 1. What do you think participating in exercise might give you?   2. If you were certain to get that benefit, would you participate?   (note: different areas of life and health; how would you feel about yourself in general) |
| Interpersonal | Sense of community   - *Welcoming* - *Social support* - *Similar others* - *Benefits of the social aspect*   The instructor’s approach   - *Encouragement and supporting enjoyment* - *Setting the right level* - *Teaching style* | Do you feel like you belong to a community?  (can you tell me a bit more about it; are there other communities you belong to?)  (note: neighbors, family, gardening club, etc)  If you were starting an exercise activity:   - Would you rather do it on your own or with others? - Would you like someone from the exercise group to contact you who you can speak to about exercise programmes which are currently available? (like an exercise buddy)? Or an instructor? - Would you like to have exercise guidance (on video or instructor) to follow? Or exercise on your own? |
| Environmental/  Organizational | Supportive surrounding (all needs)   - *Orientation* - *Elements of the session*   Affordability and convenience  Advertisement | If you were starting an exercise activity:   - Would you like to exercise outdoor or indoor? Do you have a garden? - Would you like to exercise with music or without? - Would you like to exercise with gym equipment or not? Do you have any equipment? - Would you like to monitor your achievements? (what? and how?) - Would you like regular reminders or emails about your exercise? What about other exercise opportunities? - Would you like to exercise in the morning or afternoon? For how long? - How far would you be willing to travel to join an exercise activity on normal circumstances? - How much would you pay for one session of exercise (both online and on normal circumstances)? |
| Interview guide at Phase 2 and 3  (during the lockdown and after the lockdown was eased) | | |
| Intrapersonal | General well-being and day-to day life  Engagement in physical activity and exercise (any new activities not mentioned before) | How have you been feeling since we last spoke?  What have you been doing to be physically active (if you did anything)?  Have you taken on any new activities? |
| Interpersonal | Support and contact with others | How do you keep social contact with others?  Do you receive any support or encouragement to be active? |
| Environmental | Outdoor/indoor activities | How do you spend your time during the day in the current circumstances?  How often do you go outside in the fresh air, and what do you do? |
| Policy* | Supported from organizations and the local authorities/government | Have you felt supported by any organizations, the local authorities and national Government to stay physically active over the previous few weeks (or months)? |

**Szekeres, Z. (2022). “Getting the old limbs going”: Exploring the emotional and cognitive benefits of exercise and the barriers to participation in older adults. PhD Thesis, London South Bank University https://doi.org/10.18744/lsbu.921v3

* Questions added to the semi-structured interview guide at Phase 3
