# Supplementary material for: Insights from older adults’ lived experience of physical activity and exercise during the COVID-19 lockdown in England
Source: Front Sports Act Living. 2024 Oct 31;6:1395471. doi: 10.3389/fspor.2024.1395471 (PMC11560469; doi:10.3389/fspor.2024.1395471)
Supplement: Supplementary file 2 [file Table2.docx]

# Table S2. The steps for qualitative analysis using hierarchical coding (Brooks & King, 2012; Brooks et al., 2015)

| 1. | The collected data were categorized into two groups (Active or Inactive) based on the physical activity level of the participants before the pandemic.^1^ The researchers familiarised themselves with a subset of 14 accounts (seven from inactive and seven from active older adults) by listening to the interviews and reading the full transcripts. |
| --- | --- |
| 2. | Priori themes were identified based on previous research by the research team* and the basic psychological needs theory. The a priori themes were aligned with the levels of the socio-ecological model, but proved ineffective and were redefined or removed. |
| 3. | The themes were organised into meaningful clusters and links were drawn within and between them. This step included hierarchical relationships where codes were nested into narrow themes, in turn nested into broader themes. |
| 4. | The initial coding template was identified based on six interviews (three from inactive and three from active older adults) that were the most varied from each other. In this step, the researchers compared their coding and discussed how the themes and subthemes relate to each other and developed the initial coding template together. |
| 5. | The rest of the interview transcripts were analysed using the initial template and if new codes were identified that were relevant to the research questions but did not “fit” under the initial subthemes or themes, the initial template was modified by inserting new themes or changing the existing ones. This iterative process continued until the researcher was satisfied that they reached a rich and comprehensive data representation. |
| 6. | The template was sufficient when the existing themes and subthemes covered all relevant data. At this point, the researcher presented the final template to the other members of the research team. They posed critical questions to the final template to ensure that the findings provided depth and could answer the research questions. Finally, the final template was applied to the entire dataset. |

*Note: 1. The physical activity level of each participant was assessed by using the metabolic equivalent scores based on the International Physical Activity Questionnaire - Short Form (IPAQ-SF, Sjöström et al., 2006).*

***Szekeres, Z. (2022). “Getting the old limbs going”: Exploring the emotional and cognitive benefits of exercise and the barriers to participation in older adults. PhD Thesis, London South Bank University https://doi.org/10.18744/lsbu.921v3*
