# Supplementary material for: Insights from older adults’ lived experience of physical activity and exercise during the COVID-19 lockdown in England
Source: Front Sports Act Living. 2024 Oct 31;6:1395471. doi: 10.3389/fspor.2024.1395471 (PMC11560469; doi:10.3389/fspor.2024.1395471)
Supplement: Supplementary file 3 [file Table3.docx]

# Table S3. Recommendations for the engagement of older adults in physical activity and exercise

| **Target area of recommendation** | **Recommendations** |
| --- | --- |
| **Educating** | |
| *Purpose linked with the benefits of exercise* | Older adults can be educated about the benefits of exercise for all ages and levels of mobility, especially concerning mobility and emotional wellbeing. |
| *Purpose linked with physical competence* | Older adults can be educated about establishing a routine of physical activity by using, at first, outdoor activities such as walking or gardening. |
| *Purpose linked with social interaction* | Older adults can be advised that finding a friend or family member with whom they can exercise or go on regular walks can help them to take up exercise by providing accountability. |
| *Starting points for exercise* | Older adults can be advised that exercise has the potential to break the monotony of regular daily activities. |
| **Promoting** | |
| *Being aware of the need for exercise* | Government advertisements should include positive messages about physical activity in older adults and guidance. For example, older adults can be informed about the types, intensity and amount of exercise required to gain health benefits. The information should include advice on how to fit 10-min bouts of exercise into their daily routine, so they can link the exercise with other activities they value and prioritise. |
| *Staying physically active at home* | The Government should be aware that older adults are not sufficiently physically active at home (even if they try). There should be policies to incentive older adults to go outside for physical activity. |
| *Starting points of exercise* | Promotion of the benefits of outdoor physical activity can encourage older adults to improve their physical activity. It is important to ensure the time and availability of public places where older adults can do outdoor activities safely. |
| **Supporting** | |
| *Purpose linked with interest (or priorities)* | Older adults can be supported by a healthcare professional to establish personal goals related to their health and physical function. Professionals working with older adults can assess their interests and priorities in daily life, and signpost them to local activities which meet their interests. For example, if they are interested in volunteering, encourage them to find a volunteer role, which requires some physical activity or if they like gardening, signpost them to a gardening club. |
| *Purpose linked with social interaction* | Older adults who like socializing can be signposted to team sports or group-based exercises which include an element of social gathering. |
| *Purpose linked with social interaction* | “Exercise befriending” can be provided over the phone where active older adults encourage inactive peers to do physical activity while under lockdown restrictions. |
| *Starting points for exercise (in lockdown*) | Organisations should endeavour to facilitate the contact between older adults who were previously engaged in group exercise as a way to re-engage the whole group back into group exercise sessions. |
| *Purpose linked with physical competence* | Exercise professionals working with older adults should continue tailoring exercise tasks to individual needs and physical abilities to improve confidence and provide a positive experience during exercise. |
| *Purpose linked with physical competence* | General Practitioners and Physiotherapists should give clear straightforward advice about the intensity and type of exercise that is suitable for individual older adults with long-term health conditions or health concerns. Reassurance from a healthcare professional can give confidence to older adults to take up exercise. Furthermore, regular feedback on their development from the exercise professionals can give them a sense of achievement, build their self-esteem and motivation. |
| **Enabling** | |
| *Purpose linked with physical competence/confidence* | Improving the labelling and the description of exercise programmes could help inactive older adults find a type of exercise that is most likely to suit their physical abilities and give them confidence from the start. For example, a rating system can be implemented for exercise classes from one to five where one would be exercise appropriate for people with mobility problems such as chair or fall prevention exercise and five would be appropriate for people with a high level of fitness and coordination. |
| *Convenient location* | Open, safe, and well-maintained infrastructure can enable older adults to walk more and do physical activity and exercise outdoors. Therefore, safe walking paths in parks with benches at regular intervals and hygiene facilities should be made available. |
| *Purpose linked with the benefits of exercise/interest* | Interventions should target enjoyment and social aspects as well as improving physical function to increase uptake and adherence because older adults tend to appreciate these aspects when they attend exercise programmes. For example, putting time aside for socialising immediately before or after the exercise session. |
| *Convenient location and affordability* | The portfolio of existing exercise opportunities in leisure centres, community centres and parks for older adults can be improved and the sessions must be offered cheaply or for free, preceded by “taster” sessions. |
